# P-506. Improving HIV Pre-exposure Prophylaxis (PrEP) among Transgender Individuals

**DOI:** 10.1093/ofid/ofae631.705

**Published:** 2025-01-29

**Authors:** Heather M Wang, Ruchi Biswas, Isaac Daudelin, Jonathan M Czeresnia, Michelle L Dalla-Piazza, Raquel Reyes, Shobha Swaminathan, Diana Finkel

**Affiliations:** Rutgers NJMS, Newark, New Jersey; Rutgers NJMS, Newark, New Jersey; Rutgers NJMS, Newark, New Jersey; Rutgers NJMS, Newark, New Jersey; Rutgers New Jersey Medical School, Newark, New Jersey; Rutgers NJMS, Newark, New Jersey; Rutgers New Jersey Medical School, Newark, New Jersey; Rutgers NJMS, Newark, New Jersey

## Abstract

**Background:**

Due to numerous structural factors, transgender (TG) individuals are disproportionately affected by HIV in the United States. Prioritizing PrEP for this population remains a priority. In 2020, only an estimated 3% of TG individuals were taking PrEP. However, one study showed that TG women who had access to gender-affirming care were more likely to discuss PrEP use with their healthcare provider. This study aims to evaluate PrEP prescription patterns among TG individuals seen in our integrated infectious disease and gender-affirming care practice.

**Figure:**
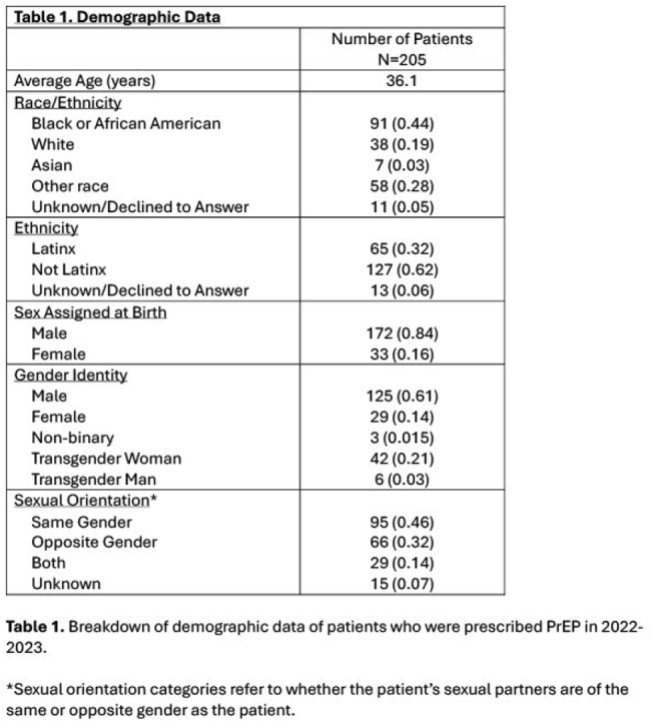

**Methods:**

We conducted a retrospective chart review for all patients who were prescribed PrEP from January 1^st^, 2022 to December 31^st^, 2023 at our institution. Data from 2017 to 2023 was collected to evaluate the frequency of PrEP prescriptions over time.

**Figure:**
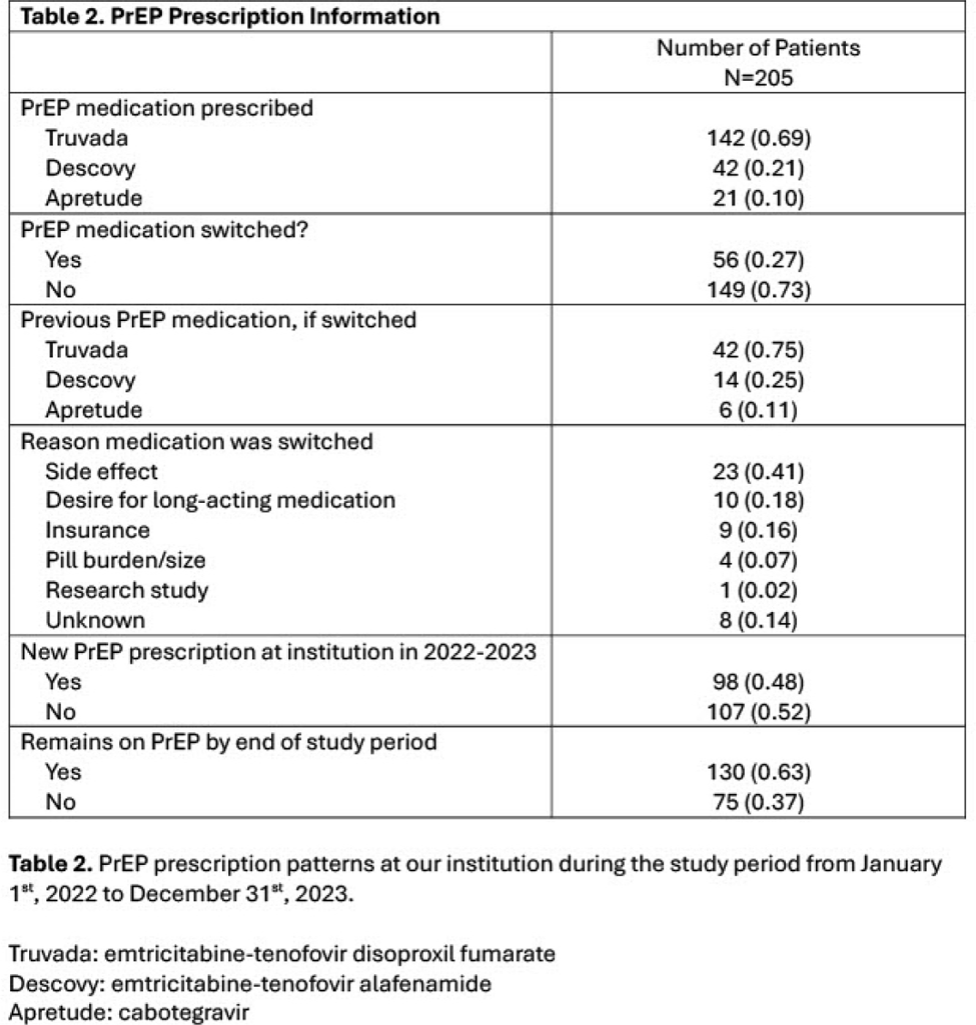

**Results:**

Two hundred and five total individuals were prescribed PrEP in 2022-2023 at our institution. Of these, 125 (61.0%) patients identified as male, 33 (16.1%) as female, 42 (20.5%) as TG female, 6 (2.9%) as TG male, and 3 (1.5%) as nonbinary. Of the 48 TG patients, 19 (39.6%), 18 (37.5%), and 13 (27.1%) were Black, Latinx, or White, respectively. Out of 48 TG patients, 32 (66.7%) were new to PrEP at our institution compared to 98 (47.8%) of all patients prescribed PrEP (p< 0.01). Thirty-four TG individuals (70.8%) remained on PrEP by the end of the study period, compared to 130/205 of all patients prescribed PrEP (63.4%; p=0.22). Linear regression indicated an increase in prescription rates in TG patients from 2017 to 2023 (R^2^ = 0.86).

**Figure:**
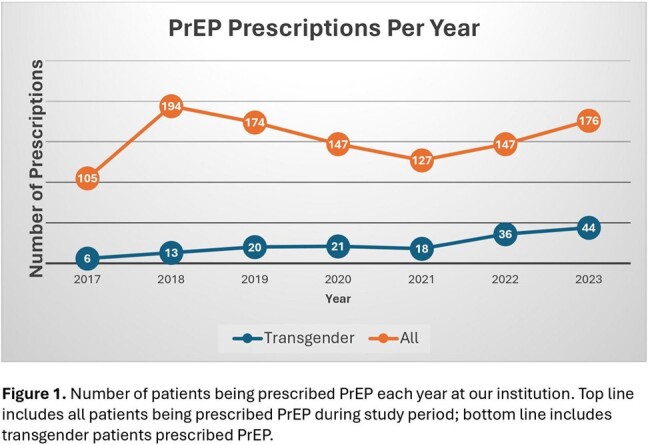

**Conclusion:**

At our institution, gender-affirming care integrated within an infectious disease practice led to higher levels of PrEP uptake and persistence for a marginalized TG patient population. Improving access to high-quality gender-affirming care is an essential component of HIV prevention programs with the goals of ending the HIV epidemic and ensuring equitable access to services.

**Figure:**
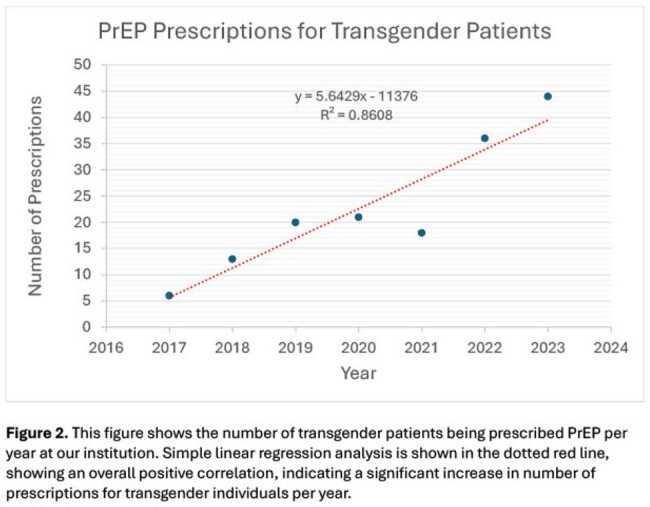

**Disclosures:**

**Shobha Swaminathan, MD**, Viiv Healthcare: Advisor/Consultant

